# Determinants of physical, psychological, and social well-being in older adults: a cross-sectional study in senior care facilities of Pakistan (2019/20)

**DOI:** 10.1186/s12877-023-04014-w

**Published:** 2023-06-05

**Authors:** Jawad Tariq, Rubeena Zakar, Mohammad Vaqas Ali, Muhammad Zakria Zakar, Amal Sajjad, Florian Fischer

**Affiliations:** 1 Department of Sociology, Forman Christian College (A Chartered University), Lahore, Pakistan; 2 Institute of Social and Cultural Studies, University of the Punjab, Lahore, Pakistan; 3 University of Poonch Rawalakot, Rawalakot, Pakistan; 4 Institute of Public Health, Charité – Universitätsmedizin Berlin, Berlin, Germany; 5 Bavarian Research Center for Digital Health and Social Care, Kempten University of Applied Sciences, Kempten, Germany

**Keywords:** Personal autonomy, Quality of care, Loneliness, Psychological well-being, Homes for the aged, Old age homes

## Abstract

**Background:**

Published research on senior care facilities in Pakistan is scarce and no large-scale study has been conducted to assess factors affecting well-being of older adults in these facilities. This study, therefore, investigated the effects of relocation autonomy, loneliness, and satisfaction with services along with socio-demographic characteristics on physical, psychological, and social well-being of older residents living in senior care facilities of Punjab, Pakistan.

**Methods:**

This cross-sectional study collected data from 270 older residents living in 18 senior care facilities across 11 districts of Punjab, Pakistan from November 2019 to February 2020 using multistage random sampling. Existing reliable and valid scales were used to collect information from older adults related to relocation autonomy (Perceived Control Measure Scale), loneliness (de Jong-Gierveld Loneliness Scale), satisfaction with service quality (Service Quality Scale), physical and psychological well-being (General Well-Being Scale), and social well-being (Duke Social Support Index). A psychometric analysis of these scales was carried out followed by three separate multiple regression analyses to predict physical, psychological, and social well-being from socio-demographic variables and key independent variables (relocation autonomy, loneliness, and satisfaction with service quality).

**Results:**

The results of multiple regression analyses showed that the models predicting physical (R^2^ = 0.579), psychological (R^2^ = 0.654), and social well-being (R^2^ = 0.615) were statistically significant (p < 0.001). Number of visitors was a significant predictor of physical (b = 0.82, p = 0.01), psychological (b = 0.80, p < 0.001), and social (b = 2.40, p < 0.001) well-being. Loneliness significantly predicted physical (b = -0.14, p = 0.005), psychological (b = -0.19, p < 0.001), and social (b = -0.36, p < 0.001) well-being. Control over relocation process significantly predicted physical (b = 0.56, p < 0.001) and psychological (b = 0.36, p < 0.001) well-being. Satisfaction with services significantly predicted physical (b = 0.07, p < 0.001) and social (b = 0.08, p < 0.001) well-being.

**Conclusion:**

Pragmatic, equitable and cost-effective interventions are needed to improve the wellbeing of older residents living in senior care facilities. Friendly behavior of mobilizing staff and adjusted residents to facilitate new residents, therapeutic interventions such as relocation support programs, reminiscence therapy and intergenerational support, and increasing their exposure and connection to the outside world, can raise their physical, psychological, and social well-being.

## Background

Demographic changes such as the increase in people aged 60 years and above have created a structural lag in many developing countries of South-East Asia like Pakistan that lack organizational structures and institutional capacities to meet the demands of older persons [1, 2]. According to the latest available statistics, Pakistan was ranked the 5th worst country for older adults [3]. There are more than 15 million people aged 60 years and above in Pakistan, and the projections show that the number will become 40 million by 2050, making them 12% of the total population [4]. A similar trend can also be observed in other South-East Asian countries bringing challenges such as the provision of healthcare facilities, transportation, jobs, recreation, housing, and increasing the number of different types of senior care facilities such as old age homes, nursing homes, assisted living facilities, retirement homes, and residential care facilities for older adults [2, 5–7]. Some of the studies from this region conducted on senior care facilities have stressed to increase the number of such facilities due to the increasing population of older adults, demand for healthcare systems, housing, changing preferences of older persons to live in such settings to avoid loneliness, abandonment of parents by the children due to changing norms of filial piety and familial care-giving, and increased abuse and neglect of older adults in home settings [1, 2, 6, 8–14]. Nevertheless, evidence also suggests that older adults may be at a higher risk of experiencing abuse and lower well-being in these senior care facilities [15–18]. The norm of filial piety encouraged children to look after aged parents but studies highlight that these norms are changing and the incidents of abuse, neglect, and forcing aged parents to vacate the house are increasing [1, 2, 12–14]. In view of these changes, the Government of Pakistan passed two laws to protect older parents and citizens, namely, *The Maintenance of Old Parents and Senior Citizens Act* of 2019 and the *Parental Protection Ordinance* of 2021 [19].

Old age homes are the common residential setting for older adults in South-East Asian countries providing accommodation, food, clothing, recreation, and basic healthcare. However, they are often stereotypically labeled as homes for older adults who have been neglected and abandoned by their families [2, 5, 20, 21]. Gerontologists and Geriatricians point out that low life satisfaction, lesser perceived control, and poor well-being may not be an outcome of aging as older adults in vulnerable settings are at a higher risk of experiencing these negative consequences as compared to other groups of older adults [1, 2, 5]. For example, studies have found that older adults who relocated to senior care facilities were at higher risk of having lower well-being and higher depression than community dwellers [22–24]. This can be theoretically explained by the continuity theory which posits that older adults seek stability and consistency in their environment instead of change. In this context, the physical environment such as one’s residence represents a sense of self, personhood, identity, meaning, and a network of embedded relations [25, 26]. This argument gains strength from the person-environment model which explains that relocation to a new setting entails detaching oneself from the meanings that the previous residence held for the person, resulting in negative consequences such as maladjustment, depression, and lower well-being [26–30]. Well-being has long been considered to be an important indicator of general health in older adults and, thus, a significant determinant of successful aging as well as an important Sustainable Development Goal (SDG3) [31, 32]. Many researchers have conceptualized well-being as a multidimensional construct and stressed to measure it along the three dimensions of physical, psychological, and social well-being [33–35]. Nevertheless, very few studies conducted on senior care facilities have focused on all three types of well-being [33, 34].

The published research literature on senior care facilities in Pakistan is scant. Very few studies have been conducted on old age homes, and the exact number of old age homes and the older adults residing in these senior care facilities in Pakistan is not known [1, 2, 5, 6, 11, 36]. Some of the studies conducted on these facilities have reported poor quality of life of older adults due to a lack of financial, structural, and social support [1, 2, 5, 6, 36]. No study was found that empirically investigated the well-being of older adults in these settings. Therefore, this study is the largest of its kind in Pakistan. The aim of this study was both empirical and normative as it statistically investigated the factors that determine the well-being of older adults in old age homes, a marginalized and neglected setting in Pakistan [1, 2, 5]. It is very important to oversee and evaluate the working of such facilities to ensure the well-being of older adults. Studies point out that voluntary relocation to senior care facilities involving choice and control followed by a supportive environment at these settings, compatibility of new residence with the previous one, and lower loneliness are the primary determinants of well-being [1, 5, 22, 25–30, 33]. Keeping in view these studies and the theoretical support provided by continuity and person-environment fit theories discussed above, the study hypothesized that higher relocation autonomy, lower loneliness, and higher satisfaction with staff and services will be related to higher well-being of older adults in old age homes of Punjab, Pakistan.

## Methods

### Study design and participants

It is a cross-sectional study. Data was collected from November 2019 to February 2020 using multistage random sampling and a structured interview schedule was used to collect data. There were a total of seven public old age homes in Punjab which were included in the study. Due to the lack of a sampling frame and absence of information related to private old age homes in Punjab, six districts were randomly selected from a total of 35 districts in Punjab. The researchers were able to locate a total of 20 private old age homes in these six districts however, permission to conduct this study was provided by administrations of 11 old age homes only. There were a total of 350 residents living in these seven public and 11 private senior care residential facilities located across 11 districts of Punjab, Pakistan, of which 290 residents gave their written consent to participate in the study. Of the remaining 60 residents, 20 refused to participate, 25 were suffering from cognitive and other physical impairments thus not being able to respond and participate, five residents were under the age of sixty, and ten were not available due to personal reasons. Of the 290 residents who were willing to participate, 20 respondents either left the interview halfway or did not respond to a lot of questions so these respondents were not included in data analysis. The final sample considered for data analysis was 270. Only those older adults were included in the study who had a minimum age of 60 years, were able to communicate, and did not have any cognitive impairment. Those residents who were not able to communicate due to any physical and/or mental disability and those who did not respond to more than 50% of the questions were excluded from the study. Cognitive impairment was not assessed using any tool (for example, mini mental state examination), but residents having some cognitive impairment as identified by administrators and staff of the old age homes were excluded from the study.

### Measures

A structured interview schedule was administered to the residents by two authors of this study who were assisted by three graduate research assistants. The three research assistants hired for this study were given two trainings and were financially compensated for their assistance. The cross-sectional survey instrument was translated from English to Urdu with the help of three experts using forward and backward translation procedure.

*Dependent variable.* The dependent variable in this study was well-being which was conceptualized along the three dimensions of physical, psychological, and social well-being. The two sub-scales to measure perceived well-being developed by Reker and Wong [37] were used to measure physical and psychological well-being, respectively. The construct to measure physical well-being had eight items (range: 0–16) and for psychological well-being six items (range: 0–12). Higher scores on both scales indicated higher well-being. Social well-being was measured by the Duke Social Support Index [38] with eleven items (range: 0–22), where higher scores showed higher social well-being. Items comprising these scales along with their psychometric properties are provided in Table 1.

**Table 1:** Psychometric properties and descriptive statistics of scales used in senior care facilities (n = 270)

*Scales*	*Loadings*	*Cronbach’s αlpha*	*Mean (SD)*	*Range*
**Relocation autonomy (AVE = 81.4%)**		0.92	4.61 (3.10)	0–8
Was it your decision to live here?	0.82			
How much input you had in the decision?	0.89			
Did you somehow influence the decision?	0.84			
Any consultations about the decision?	0.71			
**Loneliness (AVE = 59.4%)**		0.93	11.8 (6.83)	0–22
Miss pleasure of company of others?	0.87			
Often feel rejected?	0.73			
Experienced a general sense of emptiness?	0.67			
Plenty of people to lean on in case of problems?	0.66			
Have someone to talk to about daily problems?	0.70			
Circle of friends and acquaintances too limited?	0.77			
Have many people that can be trusted?	0.70			
Miss having people around you?	0.87			
Have enough people to feel close to?	0.61			
Miss having a really close friend?	0.74			
Call on friends whenever needed?	0.51			
**Service quality (AVE = 35.2%)**		0.90	32.7 (8.36)	4–50
Respectful staff?	0.78			
Polite staff?	0.71			
Questions addressed by staff?	0.65			
Comforted by staff when sad/lonely?	0.61			
Staff has time to talk about what bothers you?	0.62			
Staff knows about personal habits and tastes?	0.72			
Staff less than what is actually required?	0.58			
Staff encourages residents to establish relations?	0.72			
Staff encourages participation in activities?	0.71			
Staff too busy to answer my requests?	0.62			
Staff meets promise of coming within time?	0.50			
Not have to ask twice before something is done?	0.64			
Immediate response by staff when called?	0.70			
Staff responds instantly in medical emergency?	0.54			
Staff keeps quality of life as high as possible?	0.58			
Staff takes sincere interest in solving problems?	0.55			
Information given by staff about daily activities?	0.57			
Residents involved in decision making?	0.62			
Residents can offer feedback to concerned?	0.61			
Residents can propose suggestions?	0.71			
Residents can decide when they eat?	0.70			
Residents can decide what to eat?	0.71			
Residents decide when they want to go out?	0.79			
Residents decide which clothes they wear?	0.67			
Residents decide when to go to bed and get up?	0.60			
Residents given the privacy they want?	0.57			
**Physical well-being (AVE = 62.7%)**		0.91	9.58 (4.59)	0–16
Do not have many physical complaints?	0.67			
Do not think that I have a heart condition?	0.65			
Good appetite for food?	0.56			
Have aches and pains?	0.54			
In good shape physically?	0.65			
Is your health deteriorating?	0.58			
Do not get tired very easily?	0.66			
Can stand a fair amount of physical strain?	0.73			
**Psychological well-being (AVE = 62.1%)**		0.88	7.20 (3.39)	0–12
Is life worth living?	0.67			
Often bored?	0.51			
No one cares if I am dead or alive?	0.50			
Exciting to be alive?	0.60			
Sometimes I wish I never wake up?	0.74			
Do not seem to care about what happens to me?	0.71			
**Social well-being (AVE = 50.3%)**		0.89	5.83 (4.94)	0–22
Definite role in family and among friends?	0.58			
Family and friends understand you?	0.68			
Feel useful to family and friends?	0.58			
Feel listened to by family and friends?	0.74			
Can talk about your deepest problems?	0.53			
Know what is happening with family/friends?	0.58			
Satisfied with the relationships you have?	0.71			
Number of family members you can depend on?	0.56			
Number of times spent with someone not living here in past week?	0.65			
Number of times had telephonic conversation with friends/relatives in past week?	0.69			
Number of times attended social meetings/events in past week?	0.64			

AVE = Average Variance Extract

*Independent variables.* Relocation autonomy was measured using Perceived Control Measure [29, 39] which comprised of four items (range: 0–8). Higher score on this scale meant higher autonomy. Loneliness was measured through eleven items (range: 0–22) of the de Jong-Gierveld Loneliness Scale (DJGLS-11) [40]. A higher score on DJGLS-11 characterized higher loneliness experienced by the residents. Satisfaction with service quality was measured by the Service Quality Scale (SQS) developed by Lapré [41], which is a 26-items scale (range: 0–52) measuring satisfaction of residents along three dimensions (autonomy, engagement, and relationship with staff). Higher scores on SQS meant higher satisfaction with the services given at the facility. Items comprising these scales along with their psychometric properties are presented in Table 1.

*Covariates.* The covariates (socio-demographic variables) included in the study were gender, age, marital status, number of children and visitors, time since residence (in years), education, income, social organization (rural/urban), and residents’ visiting family/friends (yes/no).

### Data analysis

The Statistical Package for Social Sciences (SPSS), version 25, was used to generate descriptive and inferential statistics. Factor analysis using principal components and reliability analysis using Cronbach’s alpha were calculated to investigate construct validity and internal consistency of relocation autonomy, loneliness, satisfaction with service quality, and well-being scales. The Kaiser-Meyer-Ohlin (KMO) measure and p-values of Barlett’s sphericity were generated to assess sampling adequacy, correlations between items, and linearity. Descriptive statistics such as frequency, percentage, mean, standard deviation (SD), and range of obtained responses were calculated for the variables used in this study. For testing models, Cook’s and Leverage distances were used to possibly detect outliers and no outliers were found. The tolerance values for all predictor variables were close to 1 and variance inflation factor (VIF) values were under 5 so multicollinearity posed no risk [42]. The assumptions of normality, linearity, and homoscedasticity were fulfilled as assessed through histogram, normal p-p plot, and scatter plot, respectively. Three separate simple multiple regression analyses were carried out to assess the effect of predictor variables on physical, psychological, and social well-being of older adults living in senior care facilities.

### Ethics

The study received an ethics approval from the ethics committee at the Advanced Studies and Research Board at the University of the Punjab, Lahore, Pakistan (D.NO/7505/ACAD, dated October 7, 2019). The study was performed in accordance with the Declaration of Helsinki. A review of study was given and written informed consent was taken from all subjects to participate in the study. Research ethics related to confidentiality, anonymity, privacy, and safety were taken care of. The residents were informed that they would not be compensated for their participation.

## Results

### Psychometric properties and descriptive statistics of scales administered to older persons

The results showed that KMO values were greater than 0.8 in all scales (relocation autonomy, loneliness, satisfaction with service quality, and well-being scales) which suggested that linearity was present and Barlett’s p-values were less than 0.001, which suggested that the items in the relevant scales were not orthogonal. The factor loadings of all items for their relative constructs were greater than or equal to0.5 and the values for Cronbach’s alpha were greater than 0.7 which proved the construct validity and internal consistency of the scales (Table 1). The descriptive statistics (mean, SD, and range of obtained responses) of these scales are also summarized in Table 1. These descriptive statistics show that almost 50% of the residents had higher relocation autonomy, lower loneliness, and higher satisfaction with quality services. Likewise, 50% of the residents had higher physical and psychological well-being. The majority of residents had lower social well-being.

### Descriptive statistics of socio-demographic variables and their frequency distribution with respect to loneliness

The frequency distributions and percentages of socio-demographic variables and their breakdown with respect to loneliness, in addition to Chi-Square significance values, are summarized in Table 2. The mean age of older adults was 68.9 (5.79) years. More than half of residents (57.4%) were between the age of 60–69 years, of which 104 (38.5%) experienced loneliness. With respect to gender, 185 (68.5%) were males and 126 (46.6%) of them experienced loneliness in old age homes. The rate of loneliness among females was lower (24.1%). Out of 270 residents, 58.9% were widowed; among them, 43.7% reported loneliness. 238 (88.1%) residents reported that they did not have any source of income, with almost two thirds of them (62.5%) reporting loneliness. 202 (74.8%) residents said that they did not have any visitors and half of them were experienced loneliness. Likewise, 220 (81.5%) of the respondents said that they did not visit their family and friends and 60% of them reported experiencing loneliness.

**Table 2:** Distribution of socio-demographic variables with respect to loneliness in senior care facilities (n = 270)

*Variables*	*n (%)*	*Loneliness*		*X* ^*2 *^ *p-value*
		*Not lonely* *(score ≤ 6)* *n (%)*	*Lonely* *(score ≥ 7)* *n (%)*	
**Age **				0.085
60–69 years 70–79 years 80–89 years	155 (57.4) 100 (37.0) 15 (5.6)	51 (18.9) 27 (10.0) 1 (0.4)	104 (38.5) 73 (27.0) 14 (5.2)	
**Gender**				0.161
Female Male	85 (31.5) 185 (68.5)	20 (7.4) 59 (21.9)	65 (24.1) 126 (46.6)	
**Marital status**				***0.006***
Never married Currently married Widow Divorced/separated	55 (20.4) 31 (11.5) 159 (58.9) 25 (9.3)	25 (9.3) 4 (1.5) 41 (15.2) 9 (3.4)	30 (11.1) 27 (10.0) 118 (43.7) 16 (5.9)	
**Number of children**				***< 0.001***
No children 1–2 ≥ 3	96 (35.6) 67 (24.8) 107 (39.6)	40 (14.8) 26 (9.6) 13 (4.8)	56 (20.8) 41 (15.2) 94 (34.8)	
**Education**				0.407
No formal schooling Up to five years Up to eight years ≥ Matriculation	152 (56.3) 38 (14.1) 27 (10.0) 53 (19.6)	43 (15.9) 9 (3.3) 7 (2.6) 20 (7.4)	109 (40.4) 29 (10.8) 20 (7.4) 33 (12.2)	
**Any source of income**				0.792
No Yes	238 (88.1) 32 (11.9)	69 (25.6) 10 (3.7)	169 (62.5) 22 (8.2)	
**Social organization**				0.295
Rural Urban	133 (49.3) 137 (50.7)	35 (13.0) 44 (16.3)	98 (36.3) 93 (34.4)	
**Duration of residence **				0.781
Less than 1 year 1–Less than 3 years 3–Less than 5 years ≥ 5 years	43 (15.9) 117 (43.3) 47 (17.4) 63 (23.4)	10 (3.7) 37 (13.7) 14 (5.2) 18 (6.7)	33 (12.2) 80 (29.6) 33 (12.2) 45 (16.7)	
**Number of visitors**				0.072
None One Two	202 (74.8) 53 (19.6) 15 (5.6)	62 (23.0) 9 (3.3) 8 (2.9)	140 (51.8) 44 (16.3) 7 (2.7)	
**Respondent’s goes to visit**				***0.028***
No Yes	220 (81.5) 50 (18.5)	58 (21.5) 21 (7.8)	162 (60.0) 29 (10.7)	

### Multiple regression analyses estimates for models predicting physical, psychological, and social well-being

The results of multiple regression analyses (Table 3) showed that the model predicting the association of independent variables with physical well-being was statistically significant (F(13,256) = 27.10, p < 0.001, R^2^ = 0.579). Results show that one unit raise in age decreased physical well-being by 0.16 units (p < 0.001). Physical well-being was higher among residents belonging to urban areas (b = 1.14, p = 0.006). Duration of stay in the residence was a significant determinant of physical well-being as one unit increase in stay (in years) resulted in 0.21 units decrease in physical well-being (p = 0.01). The number of visitors was also a significant predictor of physical well-being as one unit increase in visitors raised physical well-being by 0.82 units (p = 0.01). Relocation autonomy, loneliness, and satisfaction with service quality were all significant predictors of physical well-being as one unit increase in relocation autonomy increased physical well-being by 0.56 units (p < 0.001), one unit increase in loneliness decreased physical well-being by 0.14 units (p = 0.005), and one unit increase in satisfaction with service quality increased physical well-being by 0.07 units (p = 0.004). The effect of remaining variables on physical well-being was statistically insignificant (Table 3).

**Table 3:** Multiple regression estimates for models predicting well-being in senior care facilities (n = 270)

*Predictors*	*Physical well-being*			*Psychological well-being*			*Social well-being*		
	*B*	*95% CI*	*p-value*	*B*	*95% CI*	*p-value*	*B*	*95% CI*	*p-value*
Age	-0.16	-0.23–-0.09	***< 0.001***	-0.01	-0.05–0.04	0.76	0.02	0.05–0.10	0.52
Gender	-0.14	-0.97–0.69	0.74	0.27	-0.29–0.83	0.34	-0.21	-1.06–0.65	0.64
Marital status	0.03	-0.41–0.46	0.91	0.09	-0.20–0.38	0.56	0.29	-0.15–0.74	0.20
Number of children	-0.21	-0.47–0.05	0.11	-0.24	-0.42–-0.07	***0.006***	0.37	0.10–0.64	***0.007***
Education	-0.13	-0.44–0.18	0.42	0.02	-0.19–0.22	0.89	-0.06	-0.38–0.26	0.72
Any source of income	-0.15	-1.46–1.17	0.83	0.21	-0.67–1.09	0.64	0.79	-0.56–2.14	0.25
Social organization	1.14	0.33–1.95	***0.006***	-0.01	-0.54–0.54	0.99	0.77	-0.06–1.60	0.07
Duration of residence	-0.21	-0.37–-0.04	***0.01***	-0.07	-0.18–0.04	0.24	-0.03	-0.20–0.14	0.70
Number of visitors	0.82	0.19–1.45	***0.01***	0.80	0.38–1.22	***< 0.001***	2.40	1.74–3.05	***< 0.001***
Respondents’ visit	0.33	-0.74–1.40	0.54	1.07	0.35–1.78	***0.004***	2.97	1.87–4.07	***< 0.001***
Relocation autonomy	0.56	0.34–0.76	***< 0.001***	0.36	0.21–0.50	***< 0.001***	0.16	-0.06–0.39	0.16
Loneliness	-0.14	-0.24–-0.04	***0.005***	-0.19	-0.25–-0.12	***< 0.001***	-0.36	-0.46–-0.26	***< 0.001***
Service quality	0.07	0.02–0.12	***0.004***	0.02	-0.01–0.05	0.23	0.08	0.03–0.13	***0.002***

Predictors ◊ Physical well-being *F*(13,256) = 27.10, p < 0.001, R^2^ = 0.579, adj. R^2^ = 0.558

Predictors ◊ Psychological well-being *F*(13,256) = 37.24, p < 0.001, R^2^ = 0.654, adj. R^2^ = 0.637

Predictors ◊ Social well-being *F*(13,256) = 31.49, p < 0.001, R^2^ = 0.615, adj. R^2^ = 0.596

The model predicting effect of independent variables on psychological well-being was statistically significant (F(13,256) = 37.24, p < 0.001, R^2^ = 0.654). Results show that a higher number of children reduces psychological well-being (b = -0.24, p = 0.006). Number of visitors and respondents’ visit to family and friends were significant predictors of psychological well-being as one unit rise in visitors raised psychological well-being by 0.80 units (p < 0.001) and one unit increase in visits resulted in 1.07 units increase in psychological well-being (p = 0.004). Relocation autonomy (b = 0.36, p < 0.001) and loneliness (b = -0.19, p < 0.001) were also significant predictors of psychological well-being. The effect of remaining variables on psychological well-being was statistically insignificant.

Table 3 shows that the model predicting relation of independent variables with social well-being was statistically significant (F(13,256) = 31.49, p < 0.001, R^2^ = 0.615). Results show that one unit raise in number of children raised social well-being by 0.37 units (p = 0.007). Number of visitors (b = 2.40, p < 0.001) and respondents’ visit to family and friends (b = 2.97, p < 0.001) were significant predictors of social well-being. Loneliness and satisfaction with service quality were also significant predictors of social well-being as one unit increase in loneliness decreased social well-being by 0.36 units (p < 0.001) and one unit increase in satisfaction with service quality increased social well-being by 0.08 units (p = 0.002). The effect of remaining variables on social well-being was statistically insignificant (Table 3).

## Discussion

The study found significant associations of various variables with the three dimensions of physical, psychological, and social well-being. Residents who had lower choice in the process of relocation had lower physical and psychological well-being and those who experienced loneliness in the facility had lower physical, psychological, and social well-being. These findings suggest that lower relocation autonomy can result in withdrawal of the residents from their new surroundings which can drive them towards isolation. For example, studies have found that lower well-being can be caused by higher levels of loneliness and one of the factors leading to higher levels of loneliness was lower relocation autonomy [29, 43]. These studies highlight that residents who had higher relocation autonomy had higher life satisfaction and lower depression as compared to those who reported lower relocation control [29, 43]. Likewise, another study found that the consequences of lower relocation autonomy were higher depression, anger, and loneliness [44]. This suggests that loneliness can act as a mediating variable between relocation autonomy and well-being and hence prospective studies should test this mediating relationship in order to understand the processes that affect well-being in old age homes. Relocating to a senior care facility with low or no control on the process can be traumatic for older persons, which can lead to a loss of interpersonal relations, meanings, identity, and purpose of life [45]. Consistent with previous studies, satisfaction with service quality significantly increased physical and social well-being [46, 47]. Studies have shown that the environment provided in such facilities like autonomy, flexibility, engagement, and respect can increase well-being of older adults [47–49]. A healthy and facilitating environment characterized by quality services can increase confidence, self-respect, and self-worth of the residents which can increase their well-being in the facility [47].

The number of people visiting older adults was a significant predictor of all three types of well-being. As the number of visitors increased, physical, psychological, and social well-being increased. This finding corroborates another finding of the study which suggested that loneliness lowered all three types of well-being in older adults living in old age homes. Researchers pointed out that higher loneliness in such facilities may arise due to a lack of social relationships and support and may thus be brief and reactive [50]. Empirical literature shows that relocation can hamper the development of new social relationships and networks and can, thereby, lower the support available to older adults leading to higher levels of loneliness and lower well-being [51]. Nevertheless, there is a likelihood that lonely older adults may actively reserve themselves from others which may lead to lower well-being [52]. This finding can be corroborated by another finding of the study which showed that those who had more visitors were less likely to experience loneliness and, therefore, had higher levels of physical, psychological, and social well-being. Similarly, those residents who visited their family and friends had higher psychological and social well-being. This finding is consistent with many studies which confirm that higher social integration and interaction in older adults resulted in higher well-being [53]. Another significant finding of the study was that a higher number of children lowered psychological well-being. This can be explained by the argument that the norms of filial piety in Eastern societies demand that children care for the parents when the latter reach old age. According to this, having more children in these societies is an assurance for the parents that their children will look after them in their late days. Such parents are more likely to face negative psychological consequences and lower well-being when children deny them care in old age, forcing them to seek shelter in old age homes [54–56].

The findings of the study should be interpreted with caution keeping in mind that it was cross-sectional, exploratory, and relational, thus making it difficult to draw causal inferences. Furthermore, the study did not attempt to explore complex interactions such as the role of loneliness and satisfaction quality as mediators of relationship between relocation and well-being of residents of old age homes.

## Conclusion

The study theoretically and empirically substantiated relationship of relocation autonomy, loneliness, and service quality with well-being by testing hypotheses derived from the propositions given by continuity and person-environment fit theories. The study will also contribute to clinical and research practices by providing psychometric validation of different scales that can be administered to older adults in old age homes and other senior care facilities. By providing pragmatic interventions, this study also expects to sensitize policymakers and relevant governments in Pakistan to address the problems of older adults living in old age homes. The study proposes pragmatic, evidence-based, and cost-effective interventions for administrators, social workers, and researchers working with older adults in senior care facilities. Interventions such as supporting new residents of senior care residential facilities to become acquainted with the facility and mobilizing staff and adjusted residents to facilitate new residents in structuring relationships can increase the physical and psychological well-being of older adults. Facilitating the residents – particularly new residents – in the adjustment process by offering relocation support programs can help to reduce relocation distress. Mobilizing the staff in these settings to provide social support through discussions, listening to problems, spending time with residents, and making them realize that they are not alone, can reduce well-being problems in senior residential care facilities. Likewise, helping residents to feel comfortable, secure, and engaged in the setting by involving them in various activities taking place in the facility can in turn develop their sense of ownership of the facility which can help to reduce loneliness and increase well-being. Increasing the social support available to the residents by (therapeutic) interventions can increase the social well-being of older adults and may even slow down the cognitive and physical decline. Administrators and managers of senior care facilities should support, facilitate, and encourage volunteers from educational institutions, community, and other welfare institutions to spend time with older adults on regular (e.g., weekly) basis. Likewise, trips may be organized for older residents of senior care facilities so that they can visit places and stay in touch with the outside world. Intergenerational social support can help the residents in the adjustment process. The administrators of old age homes as well as other senior care facilities in Pakistan, South-Asian countries, and other countries with similar contexts, keeping in view the findings of this study, can improve the well-being of older adults residing in these facilities by implementing these interventions.

## Data Availability

The dataset for this study is not publicly available due to confidentiality agreement with administrators of senior care facilities. Any individual/organization can make a reasonable request to the corresponding author of this study who will share the dataset after seeking permission from the administrators.

## Abbreviations

DJGLS: de Jong-Gierveld Loneliness Scale
KMO: Kaiser-Meyer-Ohlin
SD: Standard deviation
SPSS: Statistical Package for Social Sciences
SQS: Service Quality Scale
VIF: Variance inflation factor
